# Retinal Astrocytes: Key Coordinators of Developmental Angiogenesis and Neurovascular Homeostasis in Health and Disease

**DOI:** 10.3390/biology15020201

**Published:** 2026-01-22

**Authors:** Yi-Yang Zhang, Qi-Fan Sun, Wen Bai, Jin Yao

**Affiliations:** 1 The Affiliated Eye Hospital, Nanjing Medical University, Nanjing 210029, China; zyynjmu@stu.njmu.edu.cn (Y.-Y.Z.); sqf18066055407@stu.njmu.edu.cn (Q.-F.S.); 2 The Fourth School of Clinical Medicine, Nanjing Medical University, Nanjing 211166, China

**Keywords:** retinal astrocytes, neurovascular unit (NVU), angiogenesis, blood–retinal barrier (BRB), reactive astrogliosis, retinal vascular diseases

## Abstract

Retinal astrocytes reside mainly in the nerve fiber layer and are central to shaping retinal vessels and maintaining neurovascular balance. Derived from the optic nerve head, they spread across the inner retina to form a meshwork that both supports and instructs the emerging superficial vascular plexus. Immature astrocytes supply vascular endothelial growth factor-A(VEGF-A) to guide endothelial sprouting, while signals from growing vessels promote astrocyte maturation and strengthen the blood–retinal barrier. In disorders such as diabetic retinopathy and neovascular age-related macular degeneration, these cells show marked plasticity. Reactive astrogliosis can sustain VEGF and inflammation, favoring fragile, leaky neovessels, whereas alternative astrocyte states help reinforce barrier function and release anti-angiogenic factors. Located at the core of the neurovascular unit, astrocytes communicate continuously with endothelial cells, pericytes and neurons. This review integrates data from single-cell profiling and advanced imaging to outline astrocyte development, morphology and key signaling pathways (VEGF, PDGF, Wnt/Norrin, Eph/ephrin), and considers how tuning astrocyte polarization might be exploited to preserve retinal vascular integrity.

## Simple Summary

Many blinding eye diseases, such as diabetic retinopathy and age-related macular degeneration, are caused by abnormal blood vessel growth and fluid leakage in the retina. While current treatments primarily focus on suppressing the blood vessels themselves, this review explores a crucial but often overlooked controller of eye health: retinal astrocytes. These star-shaped support cells act as “architects” during eye development, building a structural template to guide normal blood vessel formation and sealing vessels to prevent leaks. However, this study highlights that under disease conditions, these helpful cells can switch their behavior, mistakenly fueling inflammation and destructive vessel growth instead of preventing it. We summarize current knowledge on how these cells develop, interact with neurons, and change during illness. This work is valuable to society because it suggests a new direction for medical research. By understanding how to switch these cells back to a protective state, scientists can develop novel therapies to preserve vision that go beyond the limitations of current treatments.

## 1. Introduction

Within the central nervous system (CNS), glial cells—astrocytes—are specifically believed to be involved in the maintenance of neuronal activity, maintenance of local homeostasis, and organization of the neurovascular unit (NVU). The retina, being anatomically well-defined and easily visualizable as an outpouching of the CNS, presents a uniquely powerful model for exploring neuron glial vascular interactions on account of its clear lamination and optical accessibility [1]. Müller cells and astrocytes are the two major macroglial populations within the retina [2]. Müller cells are more numerous and span all retinal layers, supporting both tissue architecture and metabolic homeostasis [3]. Astrocytes, though less abundant and mostly confined to the retinal nerve fiber layer (RNFL)/ganglion cell layer (GCL), play a critical role in vascular development and barrier formation with neuroprotection [2].

The rapid progress made in single-cell omics, genetic lineage tracing, and high-resolution imaging has broadened astrocyte research and moved glial biology into a “golden age” [3]. Although astrocyte functions in the brain, such as synaptic modulation and metabolic support, are well studied, the functional localization and morphology of astrocytes in axon rich domains like the RNFL remain poorly defined in comparison [3]. Emerging work indicates that, while being less abundant than Müller cells, retinal astrocytes coordinate many facets of retinal angiogenesis by providing a structural template, delivering guidance cues, and maintaining tissue homeostasis. Beyond furnishing a scaffold for vessel outgrowth, these cells secrete key signaling molecules, including Vascular endothelial growth factor (VEGF), PDGF, Wnt, and Eph/Ephrin family ligands, regulating endothelial behavior and shaping vascular patterning and stability.

This review synthesizes recent progress on the developmental origins, morphology, and functions of retinal astrocytes, emphasizing their multilayered regulatory roles in physiological and pathological angiogenesis. Rather than treating retinal macroglia as a single entity, we focus specifically on astrocytes as developmental templates and context-dependent regulators of the NVU. We integrate recent single-cell and spatial transcriptomic data, lineage-tracing studies, and high-resolution in vivo imaging with mechanistic work on oxygen/HIF–VEGF sensing, microglia-mediated astrocyte pruning, and astrocyte–pericyte–endothelial crosstalk. By explicitly linking developmental programs to their pathological rewiring in diabetic retinopathy (DR), retinopathy of prematurity (ROP), and neovascular age-related macular degeneration, we propose a conceptual framework that views retinal astrocytes as both scaffolds and switches for neurovascular remodeling. This framework is intended to drive testable hypotheses on disease mechanisms and to guide the design of astrocyte-targeted diagnostic and therapeutic strategies for retinal disorders.

## 2. Definition, Distribution, and Morphology of Retinal Astrocytes

### 2.1. Anatomical Localization and Network Architecture

**Figure 1:**
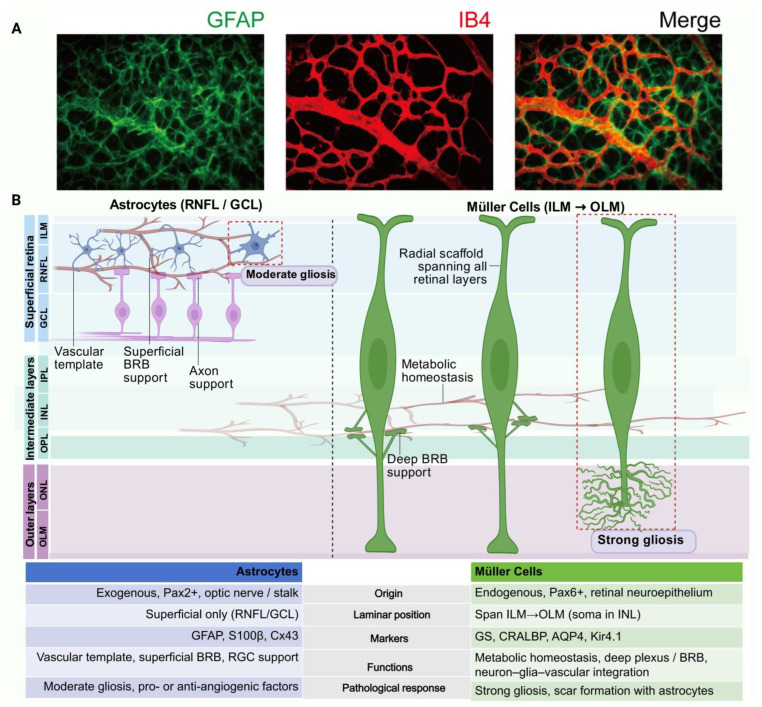
Anatomical architecture and functional specialization of retinal astrocytes. (**A**) Representative immunofluorescence images of a retinal whole mount. The astrocytic network (stained with GFAP, green) forms a continuous, two-dimensional lattice in the superficial retina that closely aligns with the vascular plexus (stained with Isolectin B4, red). This “honeycomb-like” organization provides the structural template for angiogenesis. (**B**) Schematic comparison of laminar localization. In cross-section, astrocytes are strictly confined to the superficial Nerve Fiber Layer (RNFL) and GCL, whereas Müller glia span the entire retinal depth (ILM to OLM). The table summarizes core distinctions in origin, markers, and pathological responses.

Astrocytes represent a major glial population in the CNS, pervasive throughout the brain and spinal cord, where they orchestrate synaptic activity, maintain tissue homeostasis, and couple neurovascular function [1]. Retinal astrocytes are a specialized ocular subtype found exclusively in mammals with vascularized retinas [3]. In contrast to Müller cells, which are highly abundant and span the entire retinal depth, astrocytes are less numerous and strictly confined to the RNFL and GCL (Figure 1), with their distribution closely linked to the maturation of the retinal vasculature [3].

Within these superficial layers, astrocytes associate intimately with retinal ganglion cell (RGC) axon bundles and vascular trunks, organizing into a continuous two-dimensional lattice oriented parallel to the retinal surface (Figure 1) [4,5]. Their morphology exhibits distinct species-dependent characteristics: in humans, they appear elongated or stellate [2], whereas in rodents, radial astrocytes predominate, featuring multiple symmetric processes extending from a central soma [4].

In the mature retina, this astrocytic network forms a highly organized mosaic [3,6]. Generated through contact-inhibition mechanisms, this anisotropic tiling ensures complete coverage of RGC axons and superficial vessels. Interestingly, the network topology varies by region: astrocytes at the optic nerve head display a “domain-like” organization with largely non-overlapping territories, whereas within the retina proper, their distal processes commonly overlap—a configuration that facilitates wide-field information integration and coordinated neurovascular function [7].

### 2.2. Molecular Markers and Morphological Characteristics

Glial fibrillary acidic protein (GFAP) has traditionally been used as the canonical astrocyte marker. Under physiological conditions, astrocytes express GFAP at low levels, while disease or stress induces its strong upregulation consistent with reactive gliosis [6,8]. However, relying solely on GFAP is insufficient for reliable identification [9]. In the healthy retina, the transcription factor Pax2 (Paired box gene 2) serves as a reliable nuclear marker that distinguishes astrocytes from Pax6^+^ Müller glia [3,10]. Additionally, S100β and PDGFRα are commonly used astrocyte markers in the retina, with PDGFRα frequently used to visualize the astrocyte network in retinal whole mounts [9,11]. In disease states, reactive retinal astrocytes show robust GFAP upregulation and altered intermediate filament expression, including increased vimentin and nestin, consistent with a reactive gliosis program [2,5,7].

Despite their utility for identification, these immunostaining markers label only the intermediate filament network or specific compartments, and therefore incompletely capture the form and function of astrocytes [9].

With the help of transgenic reporters, such as MORF3 mice, and three-dimensional imaging, a recent work resolved the detailed architecture of retinal astrocytes into several recurring substructures, including beaded, brush-like, sail-like, tubular, pad-like, terminal-foot, and pore-like motifs [9]. These motifs display both spatial and functional specificity: the brush-like elements dock precisely onto either RGC somata or axons, the sail-like elements drape along axon bundles, whereas the tubular elements consistently enwrap the retinal vessels [9]. Nearly all the astrocytes contact blood vessels via either specialized terminal processes or tubular projections, while forming at the same time physical interfaces with either RGC axons or somata—a configuration positioning these cells as bidirectional mediators between neural activity sensing and blood flow regulation [9]. Collectively, these anatomical features argue that the astrocytes of the retina are not passive scaffolds but active participants of the NVU, equipped with both sensing and feedback capabilities that support neurovascular coupling.

## 3. The Development of Retinal Astrocytes

### 3.1. Origins and Progenitor Cells

Retinal astrocytes have a different origin compared to that of retinal neurons and Müller glia. Extensive evidence that suggests that retinal astrocytes arise from outside the retinal neuroepithelium, migrating into the tissue from neural precursor domains [3]. The previous hypothesis involving Müller-cell transdifferentiation and in situ induction of astrocytes was ruled out through labeling, culture, and genetic evidences. In an important discovery within rat optic nerve development, a population of APCs was specified at the optic nerve/optic stalk junction that co-expressed Pax2 and A2B5—the recognized precursors of retinal astrocytes [3]. Further evidence from three converging experimental approaches support this extra-retinal origin: GFAP-tracing reveals GFAP^+^ cells in the mouse optic disc at E17 which subsequently migrate from the disc into the retina [12], and in retina–optic nerve co-culture experiments, astrocytes develop only when the optic cup stalk is retained; isolated retinas which lack this structure fail to generate astrocytes [12]. Collectively, these data indicate that the astrocytes of the retina derive from outside the neuroepithelium of the retina: they originate in the optic nerve/optic stalk region and subsequently migrate into the retina.

While the definition and morphology of astrocytes are well-characterized, their developmental origin involves a complex interplay of molecular cues. Unlike Müller glia, which share a common progenitor with retinal neurons, retinal astrocytes originate from astrocyte precursor cells (APCs) that migrate into the retina from the optic nerve. Subsequent work localizes these APCs to a specialized neuroepithelial belt encircling the cup–stalk junction, known as the “optic disc progenitor zone” [3]. Within this developmental niche, fate specification is strictly regulated by a specific molecular milieu. Key eye-primordium cues, such as Shh (Sonic Hedgehog), interact with transcription factors including Pax2 and Pax6 to drive differentiation. Specifically, these progenitors emerge from a region co-expressing the cup–stalk marker Pax2 alongside Vax2 and Raldh2, which serve as ventral-retinal markers [1,3].

They arise from a lineage different from that of the retinal neurons and Müller glia, migrating along unique spatiotemporal routes, responding to terminal positioning cues, and following distinct functional programs. The development of astrocytes is highly coordinated with the formation of the optic nerve, the extension of RGC axons, and the ingrowth of the retinal vessels. This choreography places astrocytes within the RNFL and GCL, where they form close structural and functional interfaces with RGC axon bundles and the superficial vascular plexus. This unique ontogeny underlies astrocyte participation in optic nerve–related diseases, such as glaucoma, or disorders in the development of the vessels.

### 3.2. Migration and Pattern Formation

Following specification of fate, astrocytes migrate from their extraretinal origin at the optic nerve/optic cup-stalk junction across the retinal surface to assemble a functional network. This migration is highly spatiotemporally ordered and regulated by neuronal architecture, diffusible signaling cues, and extracellular matrix (ECM) components [3]. The predominant route is radial, proceeding centrifugally along the inner limiting membrane, the retina’s innermost layer [3]. RGC axons extend from the optic disc toward the periphery before astrocyte invasion, establishing the RNFL that acts as a physical guidance template [3]. Immature astrocytes or astrocyte progenitor cells attach themselves to RGC axons and move from center to periphery. When axon guidance is perturbed, astrocytes continue to track the resultant axonal trajectories, further emphasizing RGC axons as the principal scaffold for astrocyte migration [3].

Besides axonal scaffolding, neuron-derived signals also fine-tune astrocyte migration. Retinal ganglion cell-secreted PDGF-A is a chemoattractant for the PDGFRα-positive astrocyte progenitor cells, promoting motility and directional bias [3]. Nuclear receptor Tlx (Nr2e1) regulates both the initiation and termination of astrocyte migration. The ILM (Inner limiting membrane) acts as a permissive physical substrate—there is gross disturbance in normal astrocyte distribution and trajectory due to migratory disorganization with the genetic deletion of laminin α1, a key structural component of this structure [13].

Migration of astrocytes is a species-dependent temporal window. In mice, astrocyte and astrocyte-progenitor migration starts at E17, reaching near complete pan-retinal coverage by P4–P5 [3,14]. In rats, it starts at E18, with astrocytes reaching the periphery at P8 [15]. Significantly, formation of the astrocytic network actually precedes vascularization of the retina: in mice, the vascular sprouting from the optic disc at around birth or P0–P1 occurs when the astrocytic scaffold is already partially developed—a fact that is consistent with astrocytes guiding vascular patterning [3].

Migration provides a clear morphogenetic gradient for astrocytes. The cells at the leading edge adopt simpler, often bipolar forms and express immature markers such as Pax2 and PDGFRα [3]. In contrast, the precursors, which have arrived earlier in the central retina, progressively elaborate processes and acquire the characteristic stellate morphology of mature astrocytes [15]. As migration proceeds, astrocytes tile the surface of the retina in a regular mosaic pattern [3]. This precise spatial ordering is thought to promote balanced vascular branching and appropriate capillary density during subsequent angiogenesis.

Developmentally, early extension of RGC axons establishes the primary trajectories along which astrocytes migrate. The assembled astrocytic network in turn provides structural and molecular cues that guide endothelial migration and angiogenesis, yielding a temporally ordered, nested axon–astrocyte–vessel triad that scaffolds coordinated neurovascular development [3]. Disruption of this axon templating–astrocyte migration–vascularization sequence—through RGC loss, breaches of the ILM, or perturbation of key signaling pathways—deranges astrocyte tiling and polarity and leads to abnormal vascular patterning. Such defects are implicated in the pathogenesis of ROP [16].

### 3.3. Differentiation and Maturation

After completing migration and network assembly, retinal astrocytes undergo additional differentiation to acquire their full functional phenotype. This maturation is tightly coupled to retinal vascular development and is finely regulated by vascular cues and the surrounding microenvironment [3].

Cumulative evidence indicates that vascular ingrowth is a primary driver of astrocyte terminal differentiation. Until blood vessels invade the territory, astrocytes remain in an immature state—characterized by high expression of Pax2, PDGFRα, Tlx, and VEGF-A—maintain proliferative activity, and are very sensitive to mitogens like PDGF-A [3]. With the advancement of the vascular front, the cognate astrocytes progressively leave the cell cycle, stop being responsive to PDGF-A, downregulate immature markers Pax2, Tlx, and VEGF-A, and increase mature astrocyte markers, including GFAP and S100β, thus finally acquiring a functional differentiated phenotype [3].

Oxygen acts as a critical vascular-derived signal that promotes astrocyte maturation. With increased vascularization, the local tension of oxygen activates the PHD enzymes and, by hydroxylating and degrading hypoxia-inducible factor HIF-2α [17], it promotes its hydroxylation and degradation. HIF-2α maintains the proliferative capacity in astrocyte progenitor cells and restrains their differentiation; conversely, its loss removes the brake and permits maturation. Transient hyperoxia and knockout of HIF-2α accelerate astrocyte differentiation both in mice models [3,17].

Paracrine signaling by endothelial cells (ECs) promotes astrocyte maturation. The key intermediate is leukemia inhibitory factor (LIF), secreted from ECs and acting on astrocytes to inhibit proliferation, further diminished VEGF-A expression, driving acquisition of a stable, mature phenotype [3]. The Apelin-APJ axis supports this program: Ang1-Tie2 signaling in angiogenic ECs triggers the release of Apelin from astrocytes; Apelin acts on endothelial APJ to produce LIF, creating a positive feedback loop that greatly amplifies the astrocyte-maturation signal [18]. Collectively, these pathways link vascular growth to astrocyte differentiation in developing retina.

It follows a center-to-periphery gradient in astrocyte maturation whereby regions near the optic disc, which are vascularized earliest, are also the most mature; in contrast, the angiogenic front is dominated by immature astrocyte progenitor cells or APCs [1]. As the vascular growth front progresses, the mature domain similarly expands centrifugally in a way consistent with spatiotemporal coupling, where vascular ingrowth drives astrocyte maturation [3].

The maturation of astrocytes is essential for stabilizing the retinal NVU. Mature astrocytes downregulate VEGF, thereby limiting excessive angiogenesis and preventing aberrant vascular expansion. In parallel, structural maturation—particularly the formation of perivascular end-feet—supports establishment and maintenance of the blood–retinal barrier (BRB) [19]. Together, these events form a negative feedback loop: immature astrocytes initially secrete VEGF to guide vascular ingrowth; upon vessel arrival, cues such as oxygen and LIF drive astrocyte maturation; mature astrocytes then curtail pro-angiogenic signaling and activate homeostatic support programs. This coupling ensures that vascular expansion ceases once metabolic demand is met, promoting timely transition to a stable retinal architecture and function.

### 3.4. Developmental Cell Death

During development of the retinal astrocyte network, programmed cell death (PCD) works alongside proliferation and migration to fine-tune the network’s size and layout. Carefully timed waves of normal cell removal within defined windows set the final astrocyte count, maintain even spacing, and sharpen territorial borders—together producing the mature mosaic pattern [3].

In mice, astrocyte numbers drop steeply between postnatal day 5 and 14 (P5–P14). Counts show Pax2^+^/Sox9^+^ astrocytes peak around P5 and then decline by about two-thirds to near-adult levels by P14 [3]. Lineage-tracing studies that rule out transdifferentiation or emigration point to cell death—rather than migration or fate change—as the main driver of this reduction [14].

This developmental culling is distinct from classic apoptosis. During the peak phase of cell loss, cleaved caspase-3 (CC3) is rarely detected. Furthermore, mathematical modeling indicates that the rate of astrocyte decline exceeds what can be explained by apoptosis alone [14].

Multiple lines of evidence indicate microglial phagocytosis is the primary mechanism of developmental astrocyte loss [3]. During P5–P14, microglia cluster in the RNFL, take on amoeboid shapes, and upregulate phagocytic markers (e.g., CD68, osteopontin) [14]. Confocal imaging with genetic labels (Pax2, tdTomato) captures microglia engulfing intact astrocyte cell bodies and fragments. Many targets lack hallmark apoptotic features (e.g., CC3 or marked chromatin condensation), consistent with “phagoptosis”—removal of viable or only early-injured cells before full apoptotic execution [14]. Modeling shows microglial ingestion rates can account for the rapid decline, and experimentally depleting microglia during this window produces an astrocyte surplus, underscoring their essential role [14].

This developmental pruning calibrates astrocyte number and enforces regular tiling of the mature network [3]. Because this astrocytic lattice templates developmental angiogenesis, its uniformity directly influences the layout of the vascular plexus [3]. Disrupted elimination—through microglial dysfunction or faulty “eat-me” signaling—can distort this template and derail normal vascular patterning, potentially contributing to vasculopathies such as ROP.

How microglia recognize which developing astrocytes to remove is not fully defined. Likely players include astrocytic “eat-me” cues, such as exposed phosphatidylserine, and microglial receptors like MerTK and TREM2 [3]. Pinpointing this recognition machinery will clarify fundamental mechanisms of astrocyte development and may reveal therapeutic targets for developmental retinal vascular disease.

## 4. Similarities and Differences Between Retinal Astrocytes and Müller Cells

The retina contains two main types of macroglia: astrocytes and Müller cells [8]. While Section 2 detailed the specific anatomy of astrocytes, understanding their role within the NVU requires differentiating them from Müller glia. Although they share core responsibilities—such as maintaining ionic homeostasis and supporting neuronal metabolism—their biological niches are defined by a strict division of labor rather than functional redundancy [20]. This specialization represents a finely tuned evolutionary adaptation: Müller cells act as the principal radial glia spanning the entire retinal thickness, whereas astrocytes are a specialized population introduced to govern the superficial vascular plexus [20]. Key biological distinctions are illustrated in Figure 1 and summarized in Table 1.

Spatial and Functional Complementarity The anatomical segregation outlined earlier translates into a critical “functional handover” between the two cell types. Astrocytes, forming a horizontal lattice in the RNFL/GCL, are the primary regulators of the superficial NVU and the inner blood-retina barrier at the vitreoretinal interface. In contrast, Müller cells, spanning from the ILM to the OLM, manage the metabolic and structural support for the intermediate and deep capillary plexuses as well as photoreceptors [20,21]. Thus, physiological homeostasis relies on a tiered support system where astrocytes and Müller cells manage distinct vascular and neuronal compartments.

Divergent Pathological Responses Under disease stress, these macroglia exhibit markedly different reactive profiles. Astrocytes generally display a lower threshold for activation but are more vulnerable to severe insults; in ischemic conditions, they often undergo apoptosis or form dense, fibrous glial scars that physically compartmentalize damage. Müller cells, conversely, are remarkably resilient; they typically respond via robust hypertrophy (gliosis) and upregulation of intermediate filaments without easily succumbing to cell death [2]. Furthermore, barrier dysfunction in the retina appears to be spatially compartmentalized. Astrocyte alterations preferentially affect the superficial NVU, whereas Müller cell stress is more strongly associated with disturbances of fluid homeostasis and pathology in the intermediate and deep retinal layers [2,7,20].

## 5. The Role of Retinal Astrocytes in Normal Retinal Vascularization

Retinal vascular development is a tightly regulated process that coordinates astrocyte migration and differentiation with vascular patterning and barrier assembly. Retinal astrocytes, a retina-specific macroglial subtype, arrive in the RNFL before angiogenesis. There they assemble a two-dimensional lattice that offers both a physical template and molecular guidance for endothelial ingrowth. By occupying this early niche, astrocytes help align neural and vascular development, directing vessel guidance, maturation, and BRB formation [3].

### 5.1. Astrocytes as Templates: Physical Guidance and Pattern Formation

The astrocyte network forms first and sets the spatial template for subsequent angiogenesis. Prior to the emergence of endothelial sprouts from the optic disc, astrocytes migrate into the RNFL and tile the retinal surface as a planar lattice [3]. This lattice does double duty: it provides a permissive scaffold and functions as a “path planner” that biases endothelial trajectories and branch points [3]. Loss-of-function studies underscore this role: removing astrocytes before vascular invasion prevents vessels from entering the retina, whereas ablation mid-angiogenesis does not stop peripheral advance but destabilizes new capillary loops and distorts network architecture—establishing astrocytes as indispensable organizers of retinal vascular patterning [3].

Astrocyte guidance relies on more than simple contact; it integrates adhesion and signaling:

Cadherin-mediated adhesion: R-cadherin supports transient contacts between endothelial filopodia and astrocytic processes, steering sprouting fronts and maintaining stable appositions. Fat1 cadherin further reinforces intercellular cohesion and tissue-level stability during early network assembly [18].

ECM–integrin interactions: Astrocyte-derived fibronectin and laminins engage endothelial integrins (e.g., α5β1, αvβ3, αvβ8) to guide migration and sprouting; fibronectin can potentiate VEGFR2 signaling to boost motility, while αvβ8 integrin activates TGF-β signaling to sharpen directionality and stabilize nascent vessels. Laminin–β1-integrin signaling also supports astrocyte migration and lattice formation, indirectly shaping the vascular template [18].

Additional junctional cues: N-cadherin at endothelial–glial interfaces may help stabilize contacts during maturation, though its precise contribution remains to be defined [18].

In short, the astrocyte lattice functions as a dynamic guidance system that blends spatial positioning, adhesion cues, and angiogenic signals to route endothelial sprouts with high precision. Astrocyte density and tiling tune branch density and promote the characteristic honeycomb-like capillary mesh [1,22]. Disruptions—impaired migration, network fragmentation, or faulty adhesion signaling—derail vascular patterning and can ultimately compromise visual development.

### 5.2. Vascular Stabilization and Maturation

Once the primary vascular layout is in place, astrocyte roles shift from guidance to stabilization and maturation. This includes restraining excessive angiogenesis, recruiting and supporting pericytes, and establishing/maintaining the BRB [18].

Pericyte support and vessel stabilization: Pericytes are essential for maturation, structural stability, local flow control, and barrier strength [21]. Endothelial PDGF-B recruits pericytes via PDGFRβ [21]. While astrocytes are not the main source of PDGF-B, they shape endothelial–pericyte interactions and the extracellular matrix [21]. For example, astrocyte-derived laminins can engage pericyte integrins (e.g., α2) to maintain a quiescent phenotype and prevent myofibroblast-like transitions [23]. In disease states such as DR, excessive neutrophil-derived PDGF-BB has been linked to abnormal pericyte behavior, vessel instability, and BRB breakdown [24].

BRB induction and maintenance: Through perivascular end-feet, astrocytes ensheath capillaries and release signals that promote tight-junction assembly, limiting paracellular permeability [19,21]. Disruption of astrocyte function compromises barrier integrity and increases leakage, underscoring their role in BRB biology [19]. Wnt/β-catenin signaling is central to BRB induction, driving endothelial expression of tight-junction proteins (e.g., claudin-5) and barrier-associated transporters (e.g., GLUT1) [25]. Although Wnt ligands such as Norrin are produced primarily by retinal neurons and Müller glia [26], astrocytes likely contribute via receptor-mediated responses and indirect modulation of endothelial programs.

Secreted cues for remodeling and consolidation: Astrocyte-derived signals regulate pruning, remodeling, and stabilization. The impact of astrocytic VEGF is context dependent; under hypoxic stress, it can preserve endothelial survival and maintain nascent vessels [27]. Astrocyte-derived angiopoietin-1 (Ang1) activates endothelial Tie2, strengthening integrity, reducing permeability, and enhancing endothelial–pericyte adhesion. Meteorin (METRN) from astrocytes induces thrombospondin-1/2 (TSP1/2), helping restrain excess sprouting and consolidating the capillary plexus [18].

In summary, astrocytes transition from early, template-based guidance to the later tasks of maturation and stabilization. By wrapping vessels with end-feet, coordinating endothelial–pericyte interactions, reinforcing the BRB, and releasing context-dependent cues such as VEGF, Ang1 (via Tie2), and METRN/TSP1/2, astrocytes drive the emergence of a mature, properly patterned, and stable retinal microvasculature. This role shift highlights their adaptability within the NVU and provides a mechanistic basis for durable tissue homeostasis and functional maturation of the retinal environment.

### 5.3. Regulation of Astrocyte-Related Signaling Pathways

**Figure 2:**
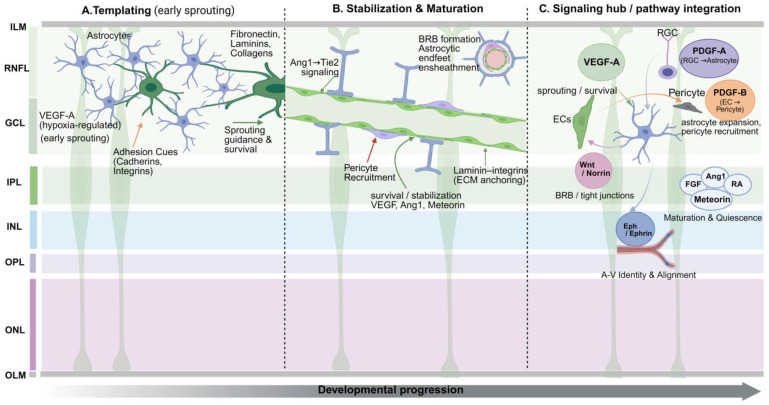
Sequential roles of retinal astrocytes in developmental angiogenesis and neurovascular maturation. (**A**) Templating. Astrocytes migrate from the optic nerve ahead of the vascular front and assemble a honeycomb scaffold; under physiological hypoxia they release VEGF-A and ECM components (fibronectin, laminins) that guide endothelial tip-cell migration and survival. (**B**) Stabilization and maturation. As vessels extend, astrocytic end-feet ensheathe capillaries and promote BRB formation, supported by Ang1–Tie2 signaling and PDGF-B–dependent pericyte recruitment. (**C**) Signaling hub. Mature astrocytes integrate neurovascular cues, expanding in response to RGC-derived PDGF-A and secreting VEGF-A, Wnt/Norrin, Eph/ephrin, and METRN to coordinate endothelial sprouting, tight-junction assembly, arteriovenous patterning, and vascular quiescence.

Astrocytes regulate retinal vascular development and homeostasis through multiple signaling pathways. The following sections synthesize the principal circuits—outlining upstream cues, key effectors, and vascular outcomes—to clarify how these pathways coordinate angiogenesis, maturation, and barrier function (Table 2). The sequential roles of astrocytes in vascular templating, stabilization, and maturation are depicted in Figure 2.

#### 5.3.1. VEGF Signaling Pathway

VEGF—especially VEGF-A—is the primary driver of retinal angiogenesis [28]. Physiological hypoxia caused by neuronal activity stabilizes HIF-1α/2α, triggering astrocytic VEGF-A release [18]. This creates a chemotactic gradient that guides VEGFR2^+^ endothelial tip cells toward the retinal periphery [3,28]. While early studies suggested astrocyte-derived VEGF was indispensable [1], astrocyte-specific knockout mice exhibit only mild expansion defects, implying compensation by Müller glia or Wnt/Norrin pathways [1]. However, astrocytic VEGF is unequivocally critical for vascular maintenance; its deletion leads to the regression of established vessels, particularly larger-caliber capillaries [27]. Thus, astrocytes provide early guidance and sustained trophic support, necessitating careful dosing of anti-VEGF therapies to avoid compromising physiological vascular survival.

#### 5.3.2. PDGF Signaling Pathway

The PDGF system coordinates neurovascular assembly via two coupled axes [23,29]:

- Neuron → Astrocyte (PDGF-A/PDGFRα): Retinal ganglion cells secrete PDGF-A to activate PDGFRα on astrocyte progenitors, promoting their proliferation, migration, and centrifugal spread [3]. Astrocytic PDGF-A overexpression expands the astrocyte field and can secondarily raise vascular density [1].
- Endothelium → Pericyte (PDGF-B/PDGFRβ): Endothelial PDGF-B recruits PDGFRβ^+^ pericytes to nascent vessels, driving mural coverage, structural stability, and reduced permeability [21,23].

Positioned at the neuro–glia–vascular interface, astrocytes integrate cues from both axes, translating neuronal activity and extracellular signals into vascular patterning, stabilization, and barrier support.

#### 5.3.3. Wnt/Norrin Signaling Pathway

Wnt/β-catenin signaling is fundamental for inducing tight junctions and establishing the BRB [25]. In the retina, Müller glia are the primary source of Norrin, the atypical Wnt ligand that activates endothelial FZD4/LRP5 receptors [25]. Although astrocytes express pathway components, their role is distinct; rather than serving as a direct source of ligands, they appear to coordinate with Müller-derived signals [1,30]. This modulation likely occurs indirectly, as astrocytes regulate local neuronal activity and metabolic demand—the upstream triggers that calibrate glial signaling output.

Functionally, this pathway operates in a critical spatiotemporal partnership with astrocytic VEGF. Early in development, the two signaling systems collaborate to stratify the vascular plexuses. In mature or pathological states, particularly where astrocytic VEGF is compromised, Wnt signaling can partially compensate to maintain endothelial survival and barrier integrity [1].

#### 5.3.4. Eph/Ephrin Signaling Pathway

The Eph/ephrin system mediates contact-dependent, bidirectional signaling essential for organizing tissue architecture and segregating retinal arteriovenous identities [31,32]. Astrocytes are integral to this patterning process, expressing a specific repertoire of isoforms, including ephrin-A1, ephrin-B2, and EphA4 [33,34]. Through heterotypic interactions with endothelial EphB4 and neuronal EphA6, astrocytes fine-tune the alignment of blood vessels with axon tracts. This contact-mediated guidance helps demarcate precise vessel–axon boundaries, contributing to the formation of specialized structures such as the foveal avascular zone.

#### 5.3.5. Auxiliary Signaling Pathway

Beyond the core angiogenic programs, astrocytes employ a diverse auxiliary toolkit to fine-tune vascular homeostasis [18,23,35,36]. To promote maturation and stability, astrocytic Ang1 activates endothelial Tie2 to reinforce junctions, a function synergistically amplified by Apelin signaling. Conversely, to restrict excessive sprouting, astrocyte-derived METRN induces thrombospondin-1/2-mediated quiescence. These stabilizing cues are balanced by FGF-driven endothelial proliferation and TGF-β-dependent sprout guidance (activated via astrocytic αvβ8 integrin). By integrating these distinct signals, astrocytes precisely calibrate vessel density and barrier properties to match local metabolic demand.

#### 5.3.6. Multipathway Integration—The Central Regulatory Role of Astrocytes

Combining the core programs (VEGF, PDGF, Wnt/Norrin, Eph/ephrin) with auxiliary pathways creates a highly interconnected, redundant, and adaptable signaling web. Within this network, astrocytes act both as sources (e.g., Ang1, Apelin, METRN) and as transducers/regulators that sculpt endothelial and mural-cell behavior. By decoding neuronal activity and integrating metabolic cues, astrocytes modulate sprouting, calibrate branching, stabilize junctions, and reinforce the BRB. This multifaceted control makes astrocytes the key integrators of neuro–glia–vascular crosstalk—aligning angiogenesis and long-term vascular homeostasis with local physiological demand.

## 6. The Role of Astrocytes in Pathological Retinal Vascularization and Disease

**Figure 3:**
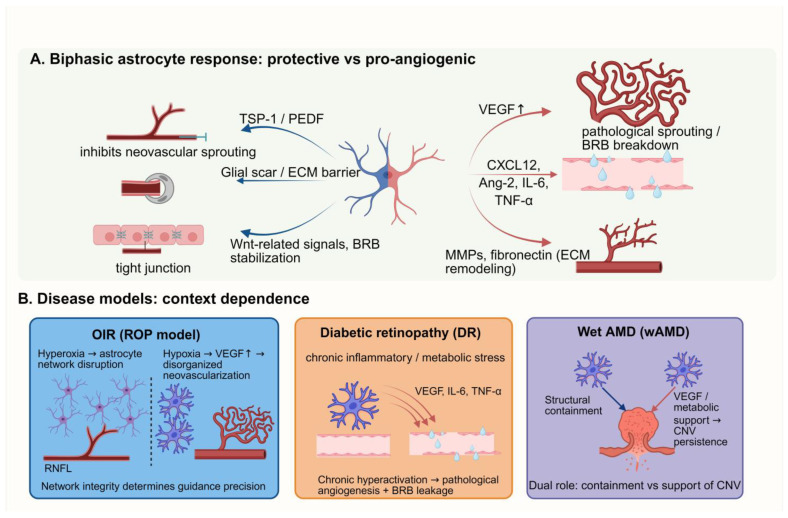
The dual and context-dependent roles of retinal astrocytes in pathological angiogenesis. (**A**) Biphasic Response. Astrocytes shift between protective and pro-angiogenic states. Protective astrocytes secrete anti-angiogenic factors (TSP-1, PEDF) and reinforce barrier integrity, whereas reactive astrocytes release VEGF, inflammatory cytokines, and MMPs, promoting pathological sprouting and BRB disruption. (**B**) Disease contexts. In OIR/ROP, hyperoxia damages the astrocytic scaffold, leading to disorganized revascularization under hypoxia. (The upward arrow “↑” indicates upregulation or increased expression). In DR, chronic inflammation drives sustained gliosis, leakage, and neovascular growth. In wet AMD, astrocytes act as a double-edged sword: they structurally encapsulate Choroidal neovascularization (CNV) lesions to limit spread, while simultaneously providing trophic support that favors vessel persistence.

Across blinding retinal diseases—DR, ROP, and wet age-related macular degeneration (wAMD)—two hallmarks stand out: pathological neovascularization (NV) and heightened vascular leak [28,37,38,39]. Retinal astrocytes play context-dependent, two-sided roles in these processes. They can protect—by stabilizing metabolism, reinforcing barriers, and supplying trophic support—yet under stress or maladaptive signaling they can also fuel disease by driving aberrant sprouting and barrier failure (Figure 3) [2,3,27].

### 6.1. Reactive Gliosis

Pathological cues such as hypoxia, hyperglycemia and mechanical strain trigger reactive astrogliosis [2,5,37]. This reactivity is not merely a cellular response but a microenvironmental remodeling force that dictates angiogenic outcomes [37,40]. Morphologically, astrocytes in the nerve fiber layer undergo hypertrophy, extending thickened processes that increase the density of the superficial glial meshwork [5,7]. At the molecular level, this transition is marked by a sharp upregulation of GFAP, re-emergence of developmental markers (e.g., nestin, vimentin), and the redistribution of connexin-43, which destabilizes the glial syncytium [2,5,41]. Crucially, a subpopulation may resume proliferation, providing the cellular mass for glial scarring [16].

Reactive gliosis is graded, not binary. Moderate activation can stabilize vessels, limit lesion spread, and protect neurons. Sustained/excessive activation promotes scarring, chronic inflammation, and VEGF-driven neovascularization—ultimately harming retinal function [8,42]. Therapeutically, tuning the “set point” and polarity of astrocyte activation is an attractive way to curb pathological NV while preserving homeostatic benefits.

### 6.2. Promotion of Pathological Angiogenesis

In ischemic retinopathies, persistent hypoxia is the main engine of pathological NV [42,43]. Under hypoxia, reactive astrocytes become active promoters via three coordinated routes:

Pathological VEGF Amplification: Under persistent hypoxia, HIF-1α/2α stabilization dramatically boosts astrocytic VEGF-A output [27,40,44]. Unlike the controlled release seen in development, this pathological excess overwhelms homeostatic signaling, directly driving the formation of disorganized, leaky neovessels and macular edema [28,45]. In Oxygen-Induced Retinopathy (OIR), surviving astrocytes within hypoxic zones serve as major sources of this dysregulated VEGF [40,46], supporting the rationale for anti-VEGF therapy in DR and wAMD [28,39].

Angiogenic–inflammatory synergy: Reactive astrocytes secrete a pro-inflammatory cocktail that exacerbates vascular instability. This includes chemokines like CXCL12 (SDF-1) to recruit endothelial progenitors, Angiopoietin-2 (Ang2) to antagonize protective Tie2 signaling, and cytokines such as TNF-α and IL-6 that amplify endothelial permeability [43,47,48].

ECM remodeling: By upregulating fibronectin and matrix metalloproteinases (MMPs), reactive astrocytes re-engineer the perivascular niche, creating physical tracks and permissive integrin signaling (e.g., α5β1) that facilitate endothelial invasion [49,50].

Under ischemic pressure, astrocytes flip from guardians to engines of pathological NV—sustaining VEGF, amplifying inflammatory/ECM signals, and accelerating neovascular growth. This rewiring exposes multiple therapeutic entry points: HIF/VEGF, Ang/Tie2, and MMP–integrin–ECM axes.

### 6.3. Inhibition or Containment of Pathological Angiogenesis

Despite their pro-angiogenic potential, astrocytes retain specific inhibitory functions that act as natural brakes on disease progression. Structurally, they coordinate with Müller glia to form glial scars that physically “fence in” pathological vessels, restricting their extension into the vitreous or deeper retina [2]. Biochemically, astrocytes produce endogenous angiostatic factors, such as TSP1 and Pigment epithelium-derived factor (PEDF), designed to dampen endothelial proliferation [28,43]; however, in severe disease states, this balance typically shifts overwhelmingly toward pro-angiogenic factors [43]. Beyond these direct vascular effects, astrocyte subpopulations attempt to bolster the blood-retina barrier (BRB) via residual Wnt/β-catenin signaling [19,51,52], although chronic stress eventually overwhelms this protective capacity [37,53]. Finally, astrocytes actively contribute to microenvironmental resolution by phagocytosing apoptotic cells and necrotic debris [2,54]. This clearance lowers Danger-Associated Molecular Patterns (DAMPs) and tempers inflammatory amplification, thereby indirectly restraining angiogenesis.

In summary, astrocytes show striking plasticity: they can drive NV or confine it. Their inhibitory toolkit—barrier formation, anti-angiogenic factors, BRB support, and inflammatory gating—offers therapeutic opportunities. Steering astrocyte polarization toward an anti-angiogenic, barrier-supportive state may enable selective, disease-modifying treatments for retinal vascular disorders.

### 6.4. Differences in Function Across Disease Models

The functional contribution of astrocytes varies significantly depending on the specific disease etiology, highlighting their context-dependent plasticity.

In the OIR model, which simulates ROP, the primary pathology is the hyperoxia-induced ablation of the immature astrocyte lattice [16,37,55]. This structural loss removes the essential guidance template, inevitably leading to chaotic, disordered revascularization during the subsequent hypoxic phase [29]. Consequently, the therapeutic imperative in ROP is not merely to inhibit VEGF, but to preserve or restore the physical integrity of the astrocytic scaffold to support orderly regrowth [3,22].

In contrast, DR presents a landscape of chronic, pro-inflammatory reactive gliosis. Here, the astrocyte network typically remains intact but becomes functionally maladaptive under the stress of hyperglycemia and oxidative stress [37,47,56]. These reactive astrocytes act as persistent sources of VEGF, IL-6, and TNF-α, actively promoting blood-retina barrier (BRB) leakage and macular edema even before NV becomes overt [4,57]. Therapeutic strategies in DR, therefore, must aim to dampen this chronic activation and bolster barrier-protective programs.

Finally, in wAMD, astrocytes play a complex structural role characterized by “containment.” Rather than guiding vessel growth, they collaborate with Müller glia to form reactive scars that physically encapsulate choroidal neovascular lesions [2,39,56]. While this scarring restricts the lesion’s expansion into the neuroretina, activated astrocytes may simultaneously secrete trophic factors that prevent the regression of pathological vessels [28,39,53]. This duality suggests that astrocytes act as structural regulators with bidirectional effects, the polarity of which remains a critical target for investigation [39,56].

## 7. Interactions Between Retinal Astrocytes and Other Cell Types

**Figure 4:**
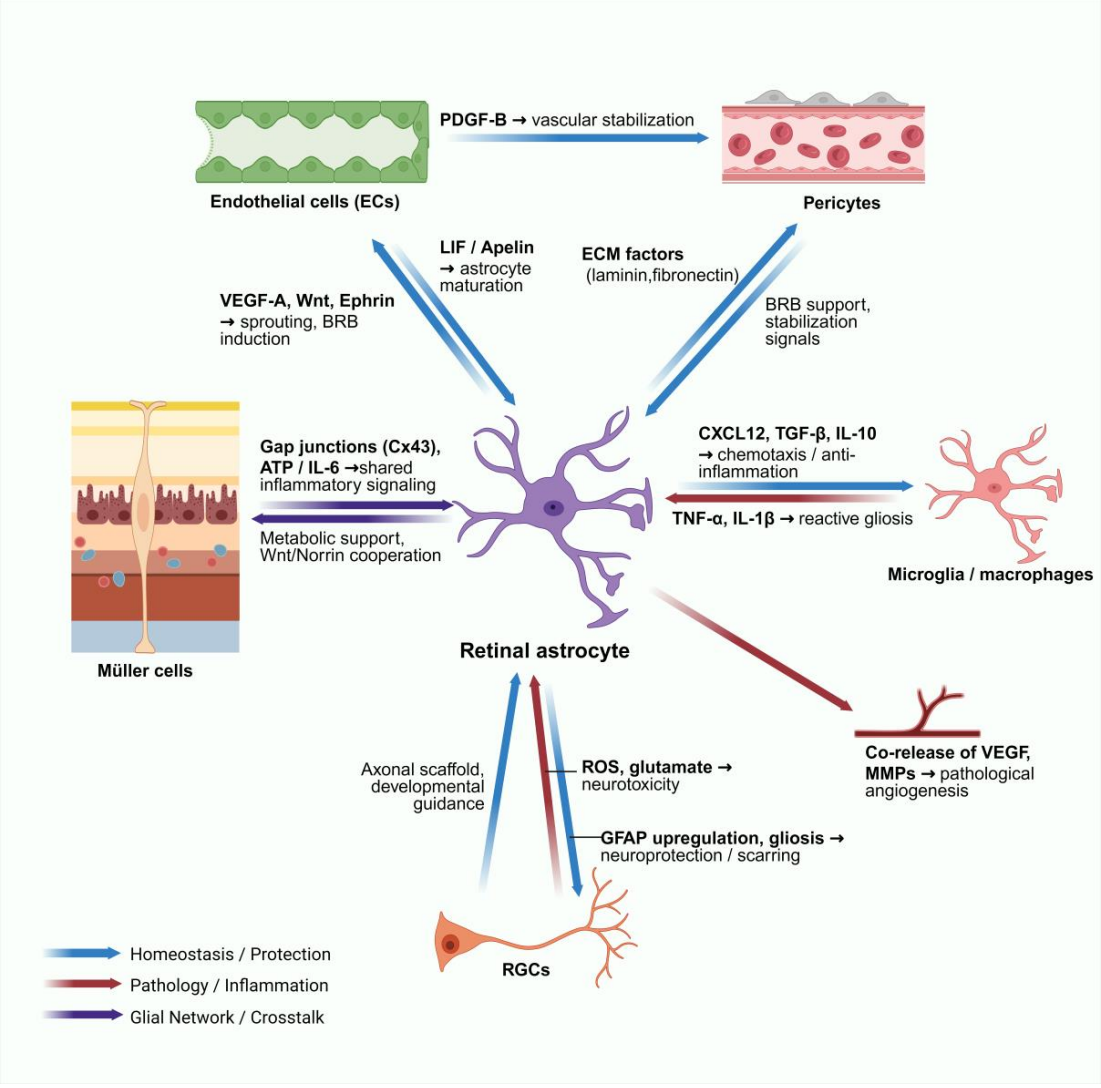
Retinal astrocytes as central hubs in the neurovascular–immune unit. Arrow colors indicate outcomes: blue, homeostatic/maturational signals; red, pathological/neurotoxic responses; purple, bidirectional glial crosstalk. Vascular interactions include endothelial LIF and Apelin driving astrocyte maturation, endothelial PDGF-B stabilizing pericytes, and astrocyte-derived VEGF-A/Wnt plus ECM deposition promoting BRB integrity. Glial and immune crosstalk involves Cx43-mediated coupling with Müller cells and reciprocal signaling with microglia (CXCL12, TGF-β versus TNF-α, IL-1β). Regarding neuronal interactions, RGC axons guide astrocyte migration, while astrocytes provide either neuroprotection or ROS/glutamate-mediated toxicity under stress. Abbreviations: BRB; Cx43; CXCL12, C-X-C motif chemokine ligand 12; ECM, extracellular matrix; IL-1β, interleukin-1 beta; LIF, leukemia inhibitory factor; PDGF, platelet-derived growth factor; RGC, retinal ganglion cell; ROS, reactive oxygen species (ROS); TGF-β, transforming growth factor-beta; TNF-α, tumor necrosis factor-alpha; VEGF, vascular endothelial growth factor.

Astrocytes sit at the hub of the retinal neurovascular–immune network. Their state is continually shaped by two-way dialogues with ECs, pericytes, Müller glia, RGCs, and microglia. These interactions drive astrocyte state transitions and, together, tune vascular homeostasis, inflammatory tone, and local metabolism—linking tissue structure, function, and signaling into a tightly coupled system. Astrocytes act as a central hub integrating signals from multiple cell types (Figure 4). The molecular mediators and functional outcomes of these interactions are comprehensively listed in Table 3.

Interactions with ECs: Astrocytes influence EC behavior through direct contact (perivascular end-feet) and paracrine signals. Astrocyte-derived cues—VEGF, Wnt/Norrin, and ephrin pathways—coordinate tip-cell sprouting, directional migration, lumen formation, and the induction of tight-junction proteins such as claudin-5 [19,27,45]. While EC-derived signals primarily target pericytes, astrocytes integrate the downstream consequences of this signaling. By sensing the maturation state of the endothelium, astrocytes adjust their end-foot coverage and polarity, closing a bidirectional loop that governs the transition from angiogenic sprouting to vascular maturation [58,59,60].

Interactions with Pericytes: Astrocytes act as critical guardians of the pericyte niche. By modulating the ECM and local PDGF signaling, astrocytes ensure pericyte retention and quiescence, which is key for endothelial–pericyte junctional integrity [23,50,60,61,62,63]. Under pathological stress, the disruption of this astrocyte-mediated support—or the transition to a reactive phenotype—compromises mural cell attachment and stability, directly contributing to vascular leakage and barrier failure [37,57].

Interactions with Müller: As the spatial complementarity of these two glial types was detailed in Section 4, their interaction here focuses on physiological coupling. Astrocytes establish direct communication with Müller cells via Connexin-43 (Cx43) gap junctions, integrating the superficial and deep retinal layers into a unified functional syncytium [41,64]. This coupling enables astrocytes to rapidly propagate metabolic and inflammatory signals (e.g., ATP, IL-6) across the entire NVU, coordinating a pan-retinal response to stress [20,64,65].

Interactions with RGCs: Developmentally, astrocytes rely on RGC axons for migration cues to populate the retina. In the mature retina, astrocytes sense neuronal activity to couple blood flow with metabolic demand [11]. However, following neuronal injury, astrocytes can switch to a reactive phenotype. While initially protective, chronically activated astrocytes may exacerbate neuronal injury through the release of glutamate, ROS, and pro-inflammatory mediators, driving a self-reinforcing glia–neuron degeneration loop [5,6,66].

Interactions with Microglia/Macrophages: Astrocytes function as critical “gatekeepers” for microglial activation [47,48,67]. By secreting CXCL12 (SDF-1), astrocytes recruit microglia to sites of injury, and release TGF-β to restrain their over-activation [48]. Conversely, astrocytes are highly sensitive to microglial-derived inflammatory signals (e.g., TNF-α, IL-1β), which can trigger their conversion into a neurotoxic phenotype. Additionally, astrocytes actively cooperate with microglia to clear cellular debris and apoptotic cells, thereby aiding in the resolution of local inflammation and reducing secondary tissue damage [2,68].

Astrocytes function as both bridges and integrators within the neuro–vascular–immune nexus. Their dialogues with ECs, pericytes, Müller cells, RGCs, and microglia combine tight anatomical coupling with diverse signaling modes, varying with context and disease state. These features explain how retinal homeostasis is maintained—and how it fails—and they highlight actionable axes for precisely tuned, cell-state-aware interventions.

## 8. Summary of Key Insights

This review has synthesized current evidence positioning retinal astrocytes not merely as passive structural scaffolds, but as dynamic, multi-functional integrators of the NVU. Three central themes emerge from the literature. First, in development, astrocytes act as the primary template for angiogenesis, guiding endothelial sprouting via oxygen-sensing HIF pathways and direct contact-mediated cues. Second, in the mature retina, they maintain the BRB and tune neurovascular coupling through a complex interplay with Müller glia, pericytes, and neurons, utilizing gap junctions to form a functional syncytium. Third, in pathology, astrocytes exhibit a “switch-like” plasticity: they can either contain damage through glial scarring and debris clearance or exacerbate disease by driving inflammation and pathological neovascularization.

Crucially, it must be acknowledged that the vast majority of these mechanistic insights—particularly those involving genetic lineage tracing and specific knockout models—derive from rodent studies. While the murine retina shares fundamental vascular and glial architectures with the human eye, it lacks key anatomical features such as the macula and the foveal avascular zone. Consequently, distinguishing between conserved fundamental biology and species-specific adaptations remains a critical filter when extrapolating these findings to human clinical pathophysiology.

## 9. Conclusions

Retinal astrocytes are far more than passive vascular templates: they function as the primary developmental scaffold, an oxygen-sensitive guidance system, and—critically—a central molecular “switch” that helps determine whether the superficial NVU progresses toward orderly maturation or pathological neovascularization. In physiological conditions, they orchestrate a precisely timed transition from pro-angiogenic immature progenitors to barrier-supporting mature cells; in disease, the same plasticity allows them to be reprogrammed into amplifiers of leaky, aberrant vessels or, conversely, into protective glial scars and endogenous anti-angiogenic factories. The emerging paradigm is therefore no longer one of astrocytes as static bystanders or mere damage responders, but of astrocytes as key regulators of retinal vascular fate. Recognizing and therapeutically redirecting this “astrocyte switch”—rather than indiscriminately blocking downstream VEGF—may offer a particularly promising path toward durable neurovascular protection in DR, ROP, and neovascular age-related macular degeneration.

## 10. Future Directions and Outstanding Questions

The past decade has transformed retinal astrocytes from “passive vascular scaffolds” to dynamic, context-dependent orchestrators of neurovascular homeostasis. Yet significant gaps remain, and addressing them could accelerate the translation of basic insights into novel diagnostics and therapies for blinding retinovascular diseases. Below we highlight four priority directions.

### 10.1. Resolving Astrocyte Heterogeneity and Subtype-Specific Functions

Single-cell and spatial transcriptomic studies have begun to delineate substantial molecular diversity within retinal astrocytes, encompassing proliferation-competent progenitors at the angiogenic front, perivascular mature subtypes, and disease-associated reactive states. A key challenge now is to align these transcriptomic profiles with their characteristic morphologies (e.g., brush-like, sail-like, tubular) and precise topological niches, using multimodal strategies such as Patch-seq, spatial proteomics, and high-resolution volume EM. Clarifying which astrocyte subtypes preferentially drive pathological versus protective responses would open the door to genuinely precision targeting, rather than indiscriminate inhibition or activation of the astrocyte population as a whole.

### 10.2. Bridging Rodent Models to Human Pathophysiology and Clinical Translation

A critical limitation is the heavy reliance on mouse models, which do not fully recapitulate human retinal anatomy. Unlike afoveate mice, humans possess a macula with a unique foveal avascular zone (FAZ) requiring distinct glial support. Morphological differences are also pronounced: while rodent astrocytes are predominantly radial, human astrocytes—especially near the optic nerve—often exhibit elongated, bipolar forms adapted to thicker axon bundles. Additionally, the human macula typically contains four vascular layers versus three in mice. Future translational success depends on validating these findings in human-relevant systems, including organoids, non-human primates, and donor tissues, to ensure clinical applicability.

### 10.3. Developing Astrocyte Polarization-Directed Therapies Beyond Anti-VEGF

Current anti-VEGF therapies, while effective, carry risks of geographic atrophy and impaired physiological angiogenesis. A more refined strategy is to pharmacologically bias astrocyte polarization toward barrier-supportive, anti-angiogenic states (e.g., enhancing thrombospondin-1/PEDF, the Meteorin–TSP1 axis, or selective HIF-2α degradation) while preserving trophic VEGF levels needed for neuronal and vascular survival. Promising candidate approaches already under preclinical investigation include small-molecule modulators of Ref-1 redox activity, integrin–ECM antagonists, and selective Wnt/FZD agonists. The challenge will be achieving sufficient retinal bioavailability and demonstrating long-term safety of shifting rather than ablating astrocyte effector pathways.

### 10.4. Astrocytes as Early Biomarkers and Imaging Targets

In experimental models of DR and OIR, reactive astrogliosis is often detectable before overt microvascular lesions appear, suggesting that glia-related readouts (e.g., GFAP upregulation, intermediate filament reorganization, connexin-43 redistribution, or characteristic secreted signatures) might provide earlier and more sensitive indicators of disease activity than conventional vascular endpoints. Recent advances in adaptive optics Optical coherence tomography (OCT), fluorescence lifetime imaging, and astrocyte-directed probes now allow non-invasive, longitudinal assessment of astrocyte morphology and activation states in vivo. When combined with liquid biopsy approaches that capture astrocyte-derived extracellular vesicles or soluble proteins, these imaging tools may ultimately support refined risk stratification and treatment monitoring at stages that precede clinically irreversible neovascular or neurodegenerative damage.

Addressing these questions will require sustained interdisciplinary collaboration among developmental biologists, vascular biologists, immunologists, and clinicians. Ultimately, shifting the therapeutic paradigm from “blocking VEGF” to “reprogramming the astrocyte switch” holds particular promise for achieving durable neurovascular protection in DR, ROP, and neovascular age-related macular degeneration.

## Figures and Tables

**Table 1:** Comparison of Retinal Astrocytes and Müller Glia.

Feature	Astrocytes	Müller Glia
Origin	Exogenous; Optic nerve/stalk progenitors	Endogenous; Retinal neuroepithelium
Lineage Markers	Pax2^+^, PDGFRα^+^, ALDH1L1^+^	Pax6^+^, VSX2^+^
Localization	Superficial only (RNFL/GCL)	Radial spanning (ILM to OLM); Soma in INL
Morphology	Stellate or elongated; Perivascular end-feet	Radial processes; Retina-wide scaffold
Quantity	Sparse	Most abundant macroglia
Typical Markers	GFAP (upregulated in reactive states), S100β, Cx43, ALDH1L1	GS, CRALBP, AQP4, Kir4.1, Cx43
Vascular Role	Forms superficial template; Guides endothelial sprouting	Supports intermediate/deep plexuses
Barrier Support	Superficial BRB	Intermediate/Deep BRB; Fluid homeostasis
Key Signals	VEGF-A, PDGF-A, Fibronectin, Eph/Ephrin	Norrin/Wnt, Metabolic factors
Pathology	Moderate gliosis; Pro- or anti-angiogenic switching	Robust gliosis; Glial scar formation

**Table 2:** Summary of Major Signaling Pathways Involving Astrocytes in Retinal Angiogenesis.

Pathway Type	Signal Source	Target Cells	Primary Function	Stage of Action
VEGF-A	Astrocytes	ECs	Bud guidance, ECs survival, anti-angiogenic regression	Early vascular development/Maturity maintenance phase
PDGF-A/PDGF-B	RGC/ECs	Astrocytes/Pericyte cells	Glial proliferation, network spreading, pericyte envelopment	Network construction phase/Stable maturation phase
Wnt/Norrin	Müllerian glial cells/Neurons	ECs	Promotes BRB formation, induces tight junctions	Maturation Stage
Eph/Ephrin	Neurons/Astrocytes	ECs/RGC	Arteriovenous separation, neurovascular space coordination	Entire Process
FGF/METRN/TSP1/2	Astrocytes/RPE	ECs/Pericytes	Auxiliary maturation, inhibition of abnormal neovascularization	Late Maturation

**Table 3:** Major cellular interactions of retinal astrocytes within the NVU.

Interaction Partners	Interaction Modalities	Representative Molecules	Functional Impact	Typical Model/Disease Context
Endothelial cells (ECs)	Terminal foot contact + mutual activation signaling	VEGF-A, Wnt, Ephrin, PDGF-B	Vascular guidance, growth, barrier formation	OIR, DR
Pericytes	ECM regulation + PDGF bridging	PDGF-A/B, Integrin, Laminin	BRB stability, vascular maturation	DR, CNV
Müller cells	Gap junctions + Paracrine coordination	Connexin43, IL-6, ATP	Inflammation regulation, structural support	DR, high-sugar model
RGCs	Anatomical guidance + state feedback interaction	ROS, glutamate, GFAP, IL-1β	Neuroprotection or damage feedback, axon guidance	Glaucoma, ischemic neuropathy
Microglia	Chemotactic activation + anti-inflammatory regulation + cooperative secretion	CXCL12, VEGF, TGF-β, TNF-α, IL-1β	Inflammatory cascade amplification/suppression, vascular remodeling, immune homeostasis	OIR, DR, Optic neuritis

## Data Availability

No new data were created or analyzed in this study. Data sharing is not applicable to this article.

## Abbreviations

The following abbreviations are used in this manuscript:

AMD	Age-related macular degeneration
Ang	Angiopoietin
BRB	Blood–retinal barrier
CC3	Cleaved caspase-3
CNS	Central nervous system
CNV	Choroidal neovascularization
CXCL12	C-X-C motif chemokine ligand 12 (also known as SDF-1)
DAMPs	Damage-associated molecular patterns
DR	Diabetic retinopathy
EC	Endothelial cell
ECM	Extracellular matrix
FAZ	Foveal avascular zone
GCL	Ganglion cell layer
GFAP	Glial fibrillary acidic protein
HIF	Hypoxia-inducible factor
ILM	Inner limiting membrane
INL	Inner nuclear layer
LIF	Leukemia inhibitory factor
METRN	Meteorin
MMP	Matrix metalloproteinase
NV	Neovascularization
NVU	Neurovascular unit
OCT	Optical coherence tomography
OIR	Oxygen-induced retinopathy
OLM	Outer limiting membrane
Pax	Paired box gene
PCD	Programmed cell death
PDGF	Platelet-derived growth factor
PDGFR	Platelet-derived growth factor receptor
PEDF	Pigment epithelium-derived factor
RGC	Retinal ganglion cell
RNFL	Retinal nerve fiber layer
ROP	Retinopathy of prematurity
ROS	Reactive oxygen species
VEGF	Vascular endothelial growth factor
wAMD	Wet age-related macular degeneration
